# Endocannabinoids and Dopamine Balance Basal Ganglia Output

**DOI:** 10.3389/fncel.2021.639082

**Published:** 2021-03-17

**Authors:** Lilach Gorodetski, Yocheved Loewenstern, Anna Faynveitz, Izhar Bar-Gad, Kim T. Blackwell, Alon Korngreen

**Affiliations:** ^1^The Mina and Everard Goodman Faculty of Life Sciences, Bar-Ilan University, Ramat Gan, Israel; ^2^The Leslie and Susan Gonda Interdisciplinary Brain Research Center, Bar-Ilan University, Ramat Gan, Israel; ^3^Department of Bioengineering, George Mason University, Fairfax, VA, United States

**Keywords:** entopeduncular nucleus, endocannabinoids, long-term plasticity, basal ganglia, dopamine

## Abstract

The entopeduncular nucleus is one of the basal ganglia's output nuclei, thereby controlling basal ganglia information processing. Entopeduncular nucleus neurons integrate GABAergic inputs from the Striatum and the globus pallidus, together with glutamatergic inputs from the subthalamic nucleus. We show that endocannabinoids and dopamine interact to modulate the long-term plasticity of all these primary afferents to the entopeduncular nucleus. Our results suggest that the interplay between dopamine and endocannabinoids determines the balance between direct pathway (striatum) and indirect pathway (globus pallidus) in entopeduncular nucleus output. Furthermore, we demonstrate that, despite the lack of axon collaterals, information is transferred between neighboring neurons in the entopeduncular nucleus *via* endocannabinoid diffusion. These results transform the prevailing view of the entopeduncular nucleus as a feedforward “relay” nucleus to an intricate control unit, which may play a vital role in the process of action selection.

## Introduction

The basal ganglia (BG) are a set of nuclei implicated in addiction (Hiroi et al., 1999), motor control (Yin, 2010), reinforcement learning (Cromwell et al., 2005; Yin et al., 2005; Lex and Hauber, 2010), and many brain disorders such as Parkinson's disease, Huntington's disease (Alexander et al., 1990; Wichmann and DeLong, 1996; Feigin et al., 2007; Kloppel et al., 2009), and Tourette syndrome (Leckman et al., 2010). The passage of information through the BG is analogous to a funnel having the striatum (Str) for a mouth, the globus pallidus (GP) as a smaller intermediate structure, and the substantia nigra pars reticulata (SNr) together with the entopeduncular nucleus (EP in rodents, homologs to the GPi in primates) as the nozzle. This funneling is characterized by a reduction in the neuronal population, which goes from (in the rodent) millions in the Str to only thousands in the EP, where the direct, indirect, and hyperdirect pathways of the BG converge (Nagy et al., 1978; Van Der Kooy and Carter, 1981; Bolam and Smith, 1992; Bevan et al., 1997; Nambu et al., 2002; Nambu, 2004; Bosch et al., 2012). The funnel like structure of the basal ganglia dictates the basic features of basal ganglia computation and dimensionality reduction (Bar-Gad and Bergman, 2001; Bar-Gad et al., 2003).

Cellular integration of excitatory (Nambu et al., 2002; Nambu, 2004; Bosch et al., 2012) and inhibitory (Smith et al., 1998) inputs from all BG pathways by individual neurons in the EP determines BG output (Kita, 2001; Kita et al., 2004; Kaneda and Kita, 2005; Kaneda et al., 2007; Bugaysen et al., 2013; Kim and Kita, 2013). A vital feature of the EP is the lack of axon collaterals between its neurons, defining it as a pure feedforward nucleus (Parent et al., 2000). In rodents and primates, direct pathway GABAergic axons from the Str connect to the dendrites of EP neurons mostly distal to the soma, while the synapses of the indirect pathway GP fibers are perisomatic (Hazrati et al., 1990; Bolam and Smith, 1992; Hazrati and Parent, 1992; Smith et al., 1998). In addition to this structural polarization, striatal projections to the EP display short-term facilitation (Sims et al., 2008; Kim and Kita, 2013), while pallidal projections to the EP display short-term depression (Kita, 2001; Sims et al., 2008; Connelly et al., 2010; Bugaysen et al., 2013).

In addition to the interplay between GABAergic inputs from the direct and indirect pathways, the EP receives glutamatergic inputs, which exhibit endocannabinoid receptor (CB1R) dependent LTD (Gorodetski et al., 2018). Interestingly, endocannabinoid (eCB) receptors are essential modulators of both inhibitory and excitatory synaptic transmission (Castillo et al., 2012; Katona and Freund, 2012). This universal function of eCBs led us to hypothesize that endocannabinoids may modulate GABAergic synaptic integration in the EP and that, as in the striatum (Kreitzer and Malenka, 2005; Freiman et al., 2006; Narushima et al., 2006a,b, 2007; Yin and Lovinger, 2006; Centonze et al., 2007; Uchigashima et al., 2007; Maccarrone et al., 2008), dopamine would modulate eCB signaling in the EP. We tested these hypotheses using whole-cell patch-clamp recording in acute brain slices. We show that synaptic integration in the EP depends on eCB release and that dopamine modulates eCB-dependent plasticity. Furthermore, we demonstrate that, despite the lack of axon collaterals, information is transferred within single EP neurons and between neighboring neurons in the EP *via* eCB diffusion.

## Materials and Methods

All procedures were approved and supervised by the Institutional Animal Care and Use Committee and followed the National Institutes of Health Guide for the Care and Use of Laboratory Animals and the Bar-Ilan University Guidelines for the Use and Care of Laboratory Animals in Research. This study was approved by the Israel National Committee for Experiments in Laboratory Animals at the Ministry of Health (permit numbers 84-12-2015 and 41-07-2016).

### Surgery and Stereotaxic Viral Injections

Five LE-Tg(DRad2-icre)1Ottc rats (RRRC Strain Acquisition Coordinator University of Missouri) (of either sex, 6–8 weeks old) were anesthetized using isoflurane, following by an I.M. injection of ketamine HCI (100 mg/kg) and xzylazine HCl (10 mg/kg). The rat's head was fixed in a stereotaxic frame, and the AAV5-EF1α-DIO-ChR2(H134R)-YFP virus (1 μl; University of North Carolina Gene Therapy Center) was injected bilaterally into the GP (AP, −0.95 mm; ML, ± 3 mm; DV, 5.75 mm) (Paxinos and Watson, 2007). The virus was injected using a syringe pump (World Precision Instruments) at a rate of 0.1 μl/min that was left in place for 10 min after injection to allow viral particle diffusion from the needle before removal. Whole-cell experiments were carried out 3 weeks after viral injection.

### *In vitro* Slice Preparation

Brain slices were obtained from Wistar rats (2–3 weeks old of either sex) as previously described (Lavian and Korngreen, 2016). Rats were anesthetized by isoflurane and killed by rapid decapitation. The brain was quickly removed and placed in ice-cold artificial cerebrospinal fluid (ACSF) containing (in mM): 125 NaCl, 2.5 KCl, 15 NaHCO_3_, 1.25 Na_2_HPO_4_, 2 CaCl_2_, 1 MgCl_2_, 25 glucose and 0.5 Na-ascorbate (pH 7.4 with 95% O_2_/5%CO_2_). In all experiments, the ACSF contained APV (50 μM) and CNQX (15 μM) to block NMDA and AMPA receptors, or gabazine (20 μM) to block GABA receptors. In some experiments, we also added AM251 (3 μm) to block CB1 receptors, sulpiride (3 μM) to block D2 receptors (D2R), R-SCH23390 (10 μM) to block D1 receptors (D1R), or quinpirole (5 μM) the D2R agonist. AM251 was dissolved in dimethyl sulfoxide (DMSO). The final concentration of DMSO was 0.15%. Thick sagittal slices (320–350 μm) were cut, using an HM 650 V Slicer (Microm International, Walldorf, Germany), at an angle of 17° to the midline to preserve functional connectivity between the STN and EP, and transferred to a submersion-type chamber where they were maintained for the remainder of the day in ACSF at room temperature. Experiments were carried out at 37°C, and the recording chamber was constantly perfused with oxygenated ACSF.

Optogenetic experiments in brain slices were performed on 6–8 weeks old Long-Evans LE-Tg (Drad2-icre)1Ottc rats 3 weeks following viral injection. To prepare brain slices, rats were deeply anesthetized using ketamine (100 mg/kg) and xylazine (10 mg/kg) and perfused transcardially with ice-cold N-methyl-D-glucamine (NMDG)–ACSF containing (in mM): 92 NMDG, 2.5 KCl, 30 NaHCO_3_, 1.25 Na_2_HPO_4_, 0.5 CaCl_2_, 10 MgSO_4_ 20 HEPES, 25 glucose, 2 thiourea and 5 Na-ascorbate (pH 7.4). The brain was quickly removed and place in the ice-cold NMDG-ACSF. Sagittal slices were cut as described above and transferred to chamber containing HEPES-ACSF containing (in mM): 92 NaCl, 2.5 KCl, 1.25 NaH_2_PO_4_, 30 NaHCO_3_, 20 HEPES, 25 glucose, 2 thiourea, 5 Na-ascorbate, 3 Na-pyruvate, 2 CaCl_2_ and 2 MgSO_4_ (pH 7.4 with 95% O_2_/5%CO_2_).

### *In vitro* Electrophysiology

Individual EP neurons were visualized using infrared differential interference contrast microscopy using an Olympus BX51WI microscope with a 60x water immersion objective (Lavian and Korngreen, 2016; Lavian et al., 2017; Gorodetski et al., 2018). Electrophysiological recordings were performed in the whole-cell configuration of the patch-clamp technique under visual control using a CCD camera (Retiga-Electro, QImaging). Recordings were obtained from the soma of EP neurons using patch pipettes (4–8 MΩ) pulled from thick-walled borosilicate glass capillaries (2.0 mm outer diameter, 0.5 mm wall thickness, Hilgenberg, Malsfeld, Germany). The standard pipette solution contained (in mM): 140 K-gluconate, 10 NaCl, 10 HEPES, 4 MgATP, 0.05 Spermin, 5 l-glutathione, 0.5 EGTA and 0.4 GTP (pH 7.2 with KOH; Sigma, St Louis, MO, USA). Under these conditions, the Nernst equilibrium potential for chloride was calculated to be −69 mV. The reference electrode was an Ag–AgCl pellet placed in the bath. Voltage signals were amplified by an Axopatch-200B amplifier or Axopatch-700B (Axon Instruments, Union City, CA, USA), filtered at 5 kHz and sampled at 20 kHz. The 10 mV liquid junction potential measured under these ionic conditions was not corrected.

Excitatory and inhibitory synaptic potentials were evoked *via* a monopolar 2–3 KΩ Narylene-coated stainless-steel stimulating microelectrode positioned in the STN, GP, or striatum. The stimulation pulse consisted of 100–800 μA biphasic currents (200 μs cathodal followed by 200 μs anodal phase). For the optogenetic experiments, inhibitory synaptic potentials were evoked *via* ChR2 activation of GP neurons by optical blue LED light (473 nm) stimulation consisted of 1–5 ms light pulses (Prizmatix). The input resistance was monitored during the experiment every few minutes. Data were excluded when the input resistance was not stable (>20% changes in the input resistance) for the entire experiment. In current clamp experiments, the EP neuron‘s membrane potential was set approximately to −60 mV by injecting positive or negative current (~0–20 pA).

### Computational Modeling

We created a multi-compartment, multi-ion channel model of EP neurons to investigate synaptic integration in response to *in vivo* like inputs. The model was implemented in the simulator MOOSE, using the moose_nerp python package, which allows declarative model specification (https://github.com/neurord/moose_nerp/tree/master/moose_nerp/ep) and simulated with a timestep of 0.001 ms.

The model includes fast and slow sodium currents, a fast and slow transient potassium current, Kv2 (non-inactivating), and Kv3 (inactivating) potassium channels, small conductance, and big conductance calcium-activated potassium channels, two hyperpolarization-activated cyclic-nucleotide gated (HCN) channels, and one high voltage-activated calcium channel. NMDA and AMPA synaptic channels and GABA synaptic channels were distributed along the dendrites. Intracellular calcium concentration was increased by influx through NMDA and calcium channels and decayed with a single time constant. Channel conductances, time constants and voltage dependence of gates, as well as membrane resistivity, axial resistivity, and capacitivity, were adjusted using the automatic parameter optimization algorithm, ajustador (available from https://github.com/neurord/ajustador), to match *in vitro* EP neuron response to hyperpolarizing and depolarizing current injection (ep032117_2_Waves available from https://github.com/neurord/waves/tree/master/EPmeasurements).

Synaptic inputs were created with a mean ISI and coefficient of variation of ISI similar to that measured *in vivo*: GPe: 29 Hz (Kita and Kita, 2011), Str: 4 Hz (for computational efficiency, each input train represents four trains firing at 1 Hz) (Kita and Kita, 2011), STN: 18 Hz (Wilson and Bevan, 2011). To measure information processing, these mean firing rates were modulated with a different frequency for each type of input (inhomogeneous Poisson process). The oscillation frequencies differed by a factor of ~3 to prevent harmonics overlapping the main frequencies. The code for spike train generation is available from https://github.com/neurord/synth_trains/. Str synaptic inputs were distributed along the dendrites, GPe inputs were distributed within 60 μm of the soma, and STN inputs were distributed everywhere. Independent trials were created by selecting a different subset from the set of spike trains and randomly selecting the location of the target synapse. Model output analysis was conducted using python 3.6; power spectra of the model output were calculated using the FFT function in NumPy, and then averaged across the set of trials.

### Analysis and Statistics

All off-line analyses of experimental data were carried out using IgorPro 7.0 (WaveMetrics; RRID:SCR_000325), Matlab R2013a (MathWorks; RRID:SCR_001622), and JASP (2019) version 0.9.2. The results for each experiment were obtained from at least three rats. The results were pooled and displayed as means ± SEM. The steady-state level of LTD and LTP was calculated as the average EPSP or IPSP amplitude 30 min after the depolarization protocols and was presented as the percentage of the average of the baseline (the first 5 min of baseline) EPSP or IPSP amplitude. A Mann-Whitney U test for paired experiments and F statistics for linear regression were used to test for significance in all the experiments.

## Results

### eCBs Mediate Long Term Changes in the Basal Ganglia Pathway

**Figure 1:**
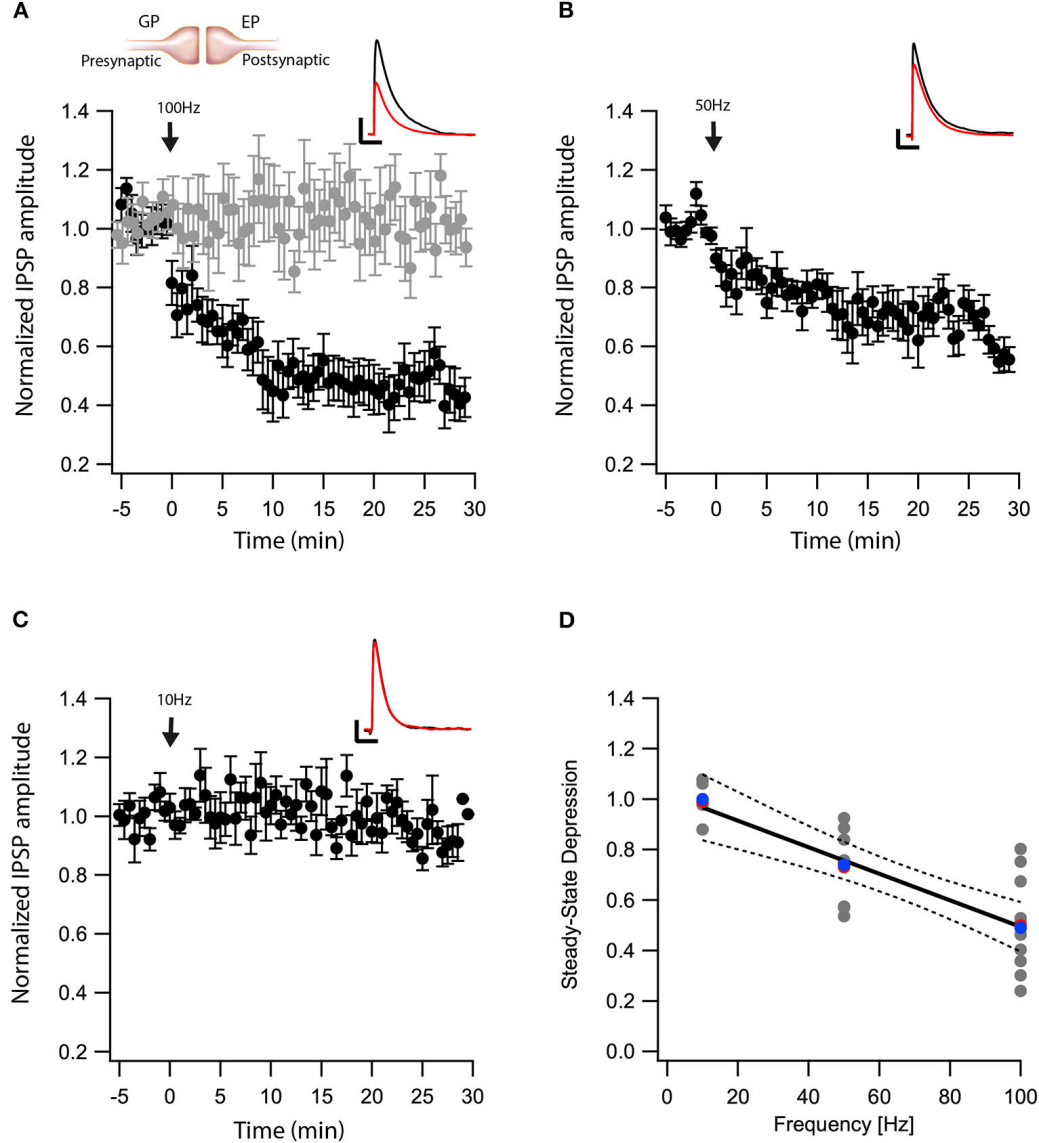
eCBs induce LTD at GP-EP synapses. **(A)** Post-HFS stimulation of 100 Hz for 10 s induced LTD at GP-EP synapses (*n* = 10). Bath application of AM251 blocked the depression at GP-EP synapses (*n* = 13) (gray). Normalized IPSP amplitudes (normalization to the mean amplitude of EPSP recorded during the baseline recordings) are plotted against time. Arrow indicates the time of induction of the post-HFS protocol, and error bars represent the SEM. Inset, traces from a representative experiment illustrating the average IPSP from the first 5 min of the baseline (black) and the final 5 min of the experiment (red). Vertical scale bar is 1 mV and the horizontal scale bar is 50 ms. **(B)** Similar to **(A)** only with a 50 Hz post-HFS stimulation (*n* = 9). **(C)** Similar to **(A)** only with a 10 Hz post-HFS stimulation (*n* = 4). **(D)** Changes in the steady-state levels of IPSP amplitudes vs. the frequency of the post-HFS protocol. The line is a linear regression of all the data. The average is marked by a red symbol and the median by a blue symbol. The 95% confidence bands are shown as dotted lines. The EP neuron‘s membrane potential was set approximately to −60 mV by injecting positive or negative current (~0–20 pA).

We recently showed that post-synaptic depolarization of neurons in the EP induces the release of endocannabinoids, which leads to long-term depression of glutamatergic input to these neurons (Gorodetski et al., 2018). Given the high density of CB1 receptors in the EP (Herkenham et al., 1990), we hypothesized that endocannabinoids might participate in long-term changes to the plasticity of other synaptic inputs to the EP. To investigate this hypothesis, we tested the effect of eCB release on GABAergic inputs to the EP from the direct) electric stimulation to the striatum) and indirect pathways (electric stimulation to the GP) of the BG. First we carried out whole-cell recordings of the membrane potential from neurons in the EP, while extracellularly stimulating in the GP using a tungsten microelectrode activating indirect pathway inputs to the EP. In the presence of CNQX and APV, brief electrical stimulation to the GP generated inhibitory synaptic responses in the EP (Figure 1A insert). Electrical stimulation in the GP can excite somata of local neurons and axons of striatal projection neurons passing through the EP. We only studied synapses displaying short-term depression (pair-pulse ratio—PPR = IPSP2/IPSP1 smaller than 1), measured using 10 pulses at 20 Hz, identifying them as GP-EP synapses of the indirect pathway (Lavian and Korngreen, 2016). After 5 min of baseline recording (103 ± 5%, *N* = 10, Figure 1A), a 10 s train of current pulses at 100 Hz was injected *via* the whole-cell electrode to the soma of the EP neuron (Gorodetski et al., 2018). This high-frequency post-synaptic stimulation protocol (post-HFS) induced robust LTD (54 ± 8%, *N* = 10, Figure 1A, *p* = 10^−5^) that was blocked by AM-251 (baseline: 102 ± 4%; after:103 ± 4%, *N* = 12, Figure 1A, *p* = 0.001). Repeating the experiment with post-HFS induction of 50 Hz resulted in a smaller LTD (baseline:100 ± 3% after:74 ± 6%, *N* = 9, Figure 1B, *p* = 0.02), whereas post-HFS using an induction frequency of 10 Hz did not affect synaptic strength (baseline:100 ± 7%; after:99 ± 8%, *N* = 4, Figure 1C, *p* = 0.802). The steady-state level of LTD displayed a monotonic dependence on the frequency of the induction protocol (Figure 1D, *F* = 34.53, *p* = 4^*^10^−6^, R^2^ = 0.58) that was similar to the monotonic dependence of the intracellular calcium concentration on firing frequency in the EP (Gorodetski et al., 2018).

**Figure 2:**
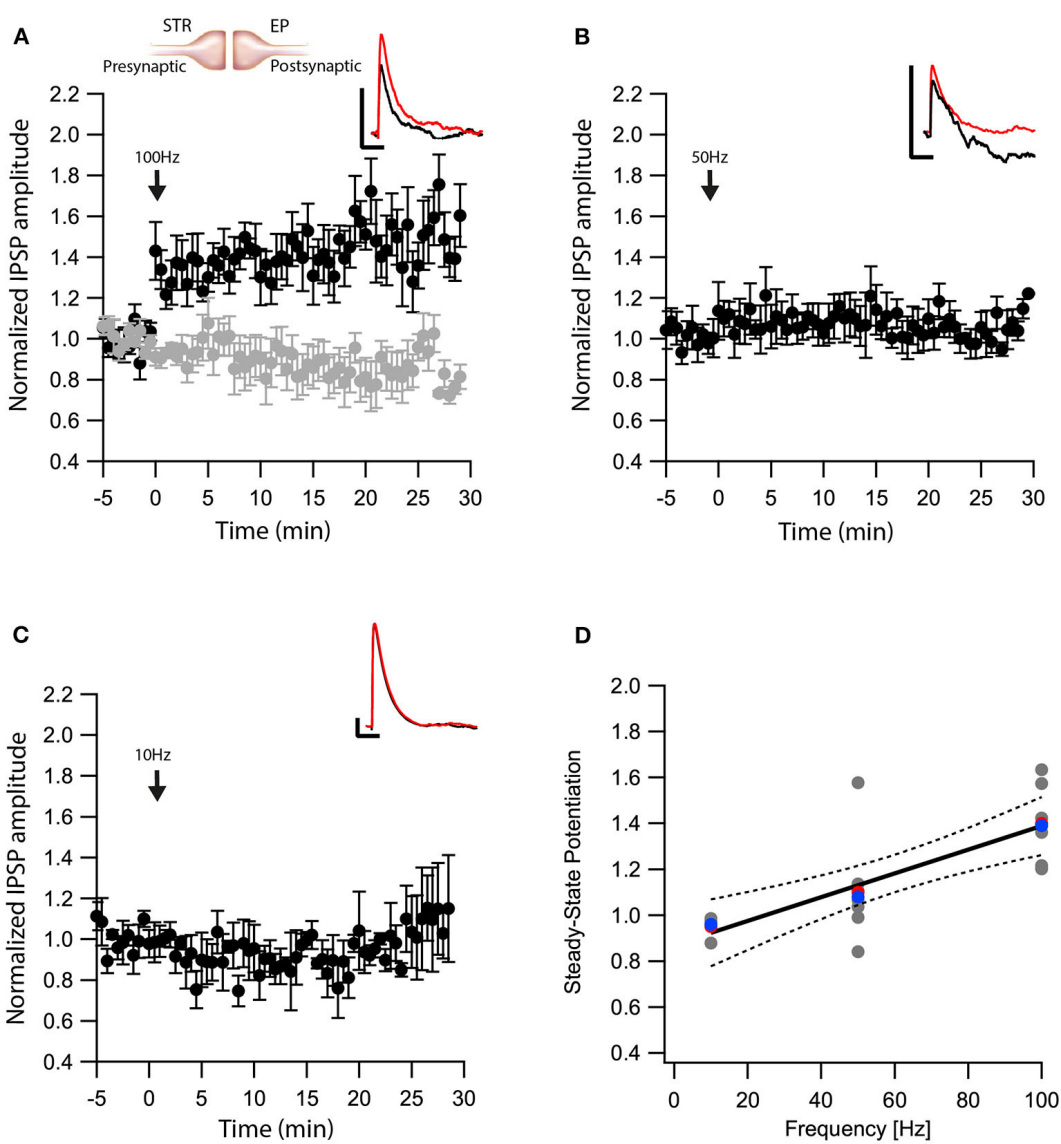
eCBs induce LTP at Str-EP synapses. **(A)** Post-HFS stimulation of 100 Hz for 10 s induced LTP at Str-EP synapses (*n* = 7) compared to the baseline response. Bath application of AM251 blocked the potentiation at Str-EP synapses (*n* = 12) (gray). Normalized IPSP amplitudes (normalization to the mean amplitude of EPSP recorded during the baseline recordings) are plotted against time. Arrow indicates the time of induction of the post-HFS protocol, and error bars represent the SEM. Inset, traces from a representative experiment illustrating the average IPSP from the first 5 min of the baseline (black) and the final 5 min of the experiment (red). Vertical scale bar is 1 mV and the horizontal scale bar is 50 ms. **(B)** Similar to **(A)** only with a 50 Hz post-HFS stimulation (*n* = 4). **(C)** Similar to **(A)** only with a 10 Hz post-HFS stimulation (*n* = 6). **(D)** Changes in the steady-state levels of IPSP amplitudes vs. the frequency of the post-HFS protocol. The line is a linear regression of all the data. The average is marked by a red symbol and the median by a blue symbol. The 95% confidence bands are shown as dotted lines. The EP neuron‘s membrane potential was set approximately to −60 mV by injecting positive or negative current (~0–20 pA).

Next, we tested whether eCBs modulated direct pathway input to the EP. We carried out whole-cell recordings of the membrane potential from EP neurons while applying electrical stimulation to the striatum. In the presence of CNQX and APV, brief electrical stimulation to the striatum generated inhibitory synaptic responses in EP neurons (Figure 2A insert). Synapses from striatal projection neurons onto EP neurons display short-term facilitation (Lavian and Korngreen, 2016). Before performing plasticity experiments, we verified this feature of direct-pathway synapses by applying a train of 10 stimulations at 20 Hz to the striatum. We identified synapses displaying only short-term facilitation as Str-EP synapses, which were further investigated. After 5 min of baseline recording (99 ± 6%, *N* = 7, Figure 2A), we stimulated the neuron with a 100 Hz post-HFS for 10 s. Contrary to the GP-EP synapse, this protocol induced LTP of the direct pathway input to the EP (144 ± 11%, *N* = 7, Figure 2A, *p* = 0.0044) that was blocked by AM251 (baseline:100 ± 4%; after:89 ± 8%, *N* = 12, Figure 2A, *p* = 10^−5^). Unlike the GP-EP synapse, a 50 Hz post-HFS protocol generated a small, not significant change in synaptic plasticity (baseline:102 ± 9%; after:107 ± 7%, *N* = 8, *p* = 0.003, Figure 2B). Finally, a 10 Hz post-HFS protocol had no long-term effect on synaptic plasticity in Str-EP synapses (baseline:100 ± 7%; after:99 ± 10%, *N* = 4, Figure 2C, *p* = 0.44), similar to the GP-EP synapses. The monotonic dependence of LTP on post-synaptic firing (Figure 2D, *F* = 20.49, *p* = 0.0003, R^2^ = 0.547) was similar to the monotonic dependence of the intracellular calcium concentration on firing frequency in the EP (Gorodetski et al., 2018).

**Figure 3:**
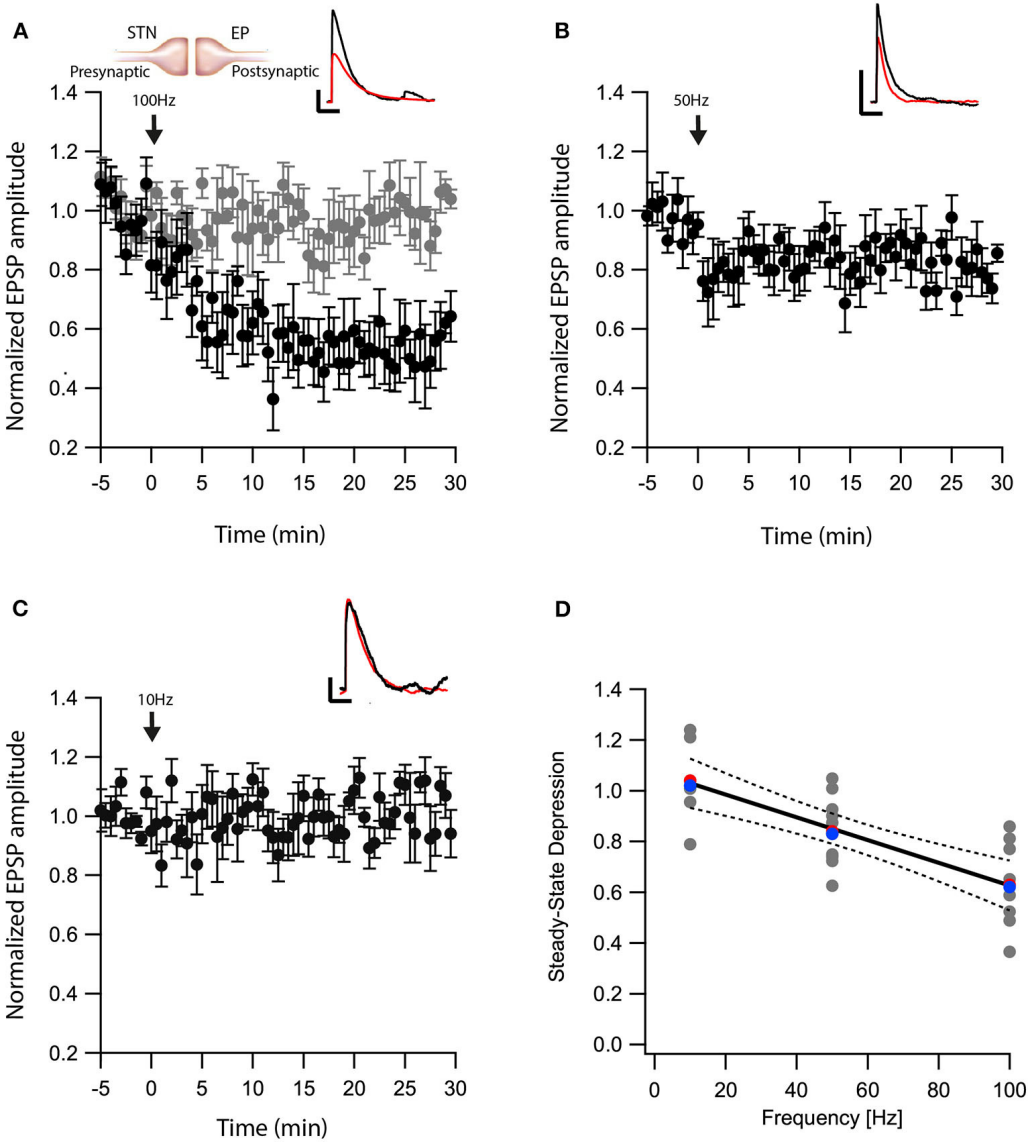
eCB induce LTD at STN-EP synapses. **(A)** Post-HFS stimulation of 100 Hz for 10 s induced LTD at STN-EP synapses (*n* = 8) compared to the baseline response. Bath application of AM251 blocked the depression at STN-EP synapses (*n* = 10) (gray). Normalized EPSP amplitudes (normalization to the mean amplitude of EPSP recorded during the baseline recordings) are plotted against time. Arrow indicates the time of the post-HFS protocol, and error bars represent the SEM. Inset, traces from a representative experiment illustrating the average EPSP from the first 5 min of the baseline (black) and the final 5 min of the experiment (red). Vertical scale bar is 1 mV and the horizontal scale bar is 50 ms. **(B)** Same as in **(A)** only with a 50 Hz post-HFS stimulation (*n* = 13). **(C)** Same as in A only with a 10 Hz post-HFS stimulation (*n* = 9). **(D)** Changes in the EPSPs amplitude vs. the frequency of the depolarization of the EP neurons. The line is a linear regression of all the data. The average is marked by a red symbol and the median by a blue symbol. The 95% confidence bands are shown as dotted lines. The EP neuron‘s membrane potential was set approximately to −60 mV by injecting positive or negative current (~0–20 pA).

Another nucleus sending projections to the EP is the STN. The glutamatergic synapses between the STN-EP, part of the hyper-direct pathway, convey activity from the cortex to the EP. We previously showed that post-synaptic depolarization induces eCB dependent synaptic plasticity of glutamatergic input to the EP in the hyperdirct pathway (Gorodetski et al., 2018), the frequency dependence of this plasticity is unknown. Thus, we applied similar post-HFS protocols as described above while stimulating extracellularly in the STN in the presence of gabazine in order to record glutamatergic synaptic transmission in isolation. Similar to the results obtained at the GP-EP synapse (Figure 1), a post-HFS protocol at 100 Hz generated robust LTD (baseline:101 ± 7%; after:60 ± 10%, *N* = 7, Figure 3A, *p* = 0.04) that was blocked by AM251 (baseline:103 ± 5%; after:98 ± 8%, *N* = 10 Figure 3A, *p* = 0.0002), post-HFS at 50 Hz generated a smaller LTD (baseline:97 ± 6%; after:83 ± 8%, *N* = 11, Figure 3B, *p* = 0.002), and post-HFS at 10 Hz APs did not generate a significant synaptic plasticity (baseline:101 ± 8%; after:101 ± 9%, *N* = 7, Figure 3C, *p* = 0.428). Glutamatergic synaptic plasticity was monotonically dependent on the frequency of the post-synaptic stimulation (Figure 3D, *F* = 28.69, R^2^ = 0.544).

### eCBs Modulate Plasticity in Neighboring Neurons

**Figure 4:**
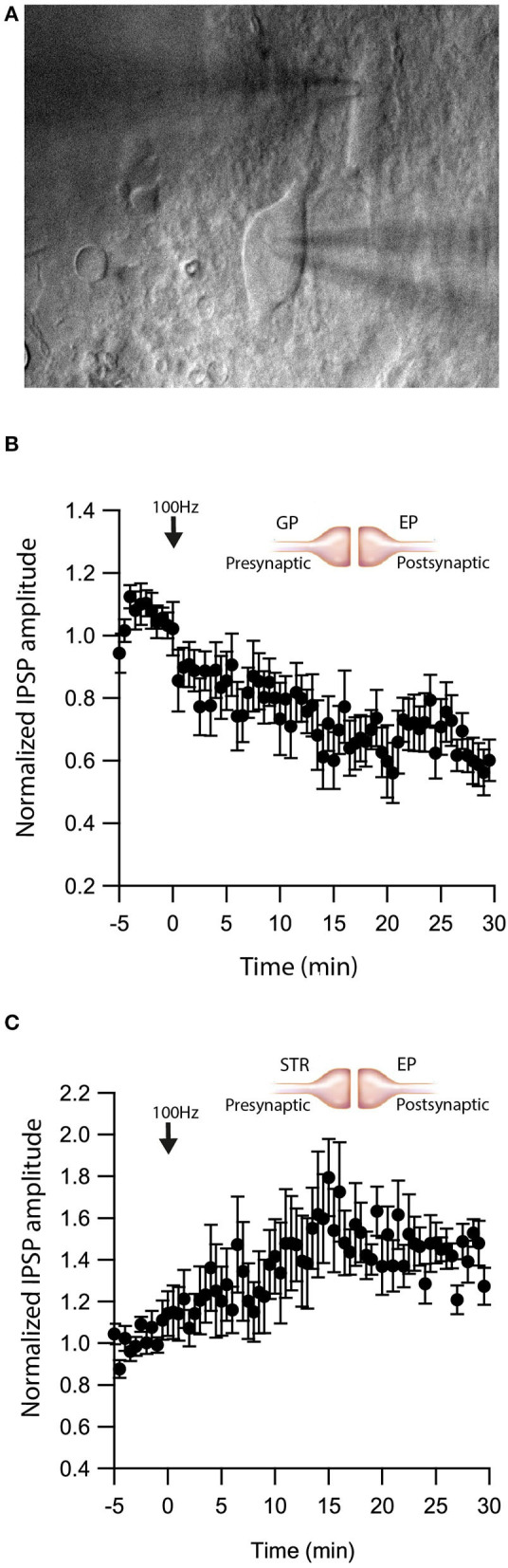
eCBs induce synaptic plasticity in neighboring neurons. **(A)** An image of a paired recording from two neurons in the EP. **(B)** Post-HFS at 100 Hz for 10 s to one neuron induced LTD at GP-EP synapses of the non-stimulated neuron (*n* = 13). Normalized IPSP amplitudes (normalization to the mean amplitude of IPSP recorded during the baseline recordings) are plotted against time. Arrow indicates the time of induction of the depolarization protocol, and error bars represent the SEM. **(C)** Post-HFS at 100 Hz for 10 s to one neuron induced LTP at Str-EP synapses of the non-stimulated neuron (*n* = 5).

As retrograde messengers, eCBs diffuse and affect synapses in surrounding neurons (Kreitzer and Regehr, 2001; Maejima et al., 2001; Ohno-Shosaku et al., 2001; Yanovsky et al., 2003; Zhu, 2005). To test whether this lateral transfer of information occurs in the EP, we performed paired recordings from neighboring neurons (inter-somatic distance <40 μm) in the EP (Figure 4A) and measured eCB induced plasticity (Figure 4). We placed an extracellular stimulating electrode in the GP and applied a single stimulus to test for a synaptic connection to at least one of the neurons. We stimulated the soma of one of the neurons with a 10 s post-HFS at 100 Hz and measured changes in synaptic strength of inputs to the unstimulated neuron (Figure 4B).

Post-HFS of one neuron generated LTD in the GP-EP GABAergic synapse on the unstimulated neuron (baseline:101 ± 7%; after:73 ± 8%, *N* = 13, Figure 4B, *p* = 0.0012). Because concentration decreases with distance for diffusing molecules, such a mechanism suggests that the LTD would be lower in more distant neurons; however, we did not detect a correlation between the magnitude of LTD and the distance between the two somata. Placing the stimulating electrode in the Str produced a similar trend, eCB release by post-HFS in one neuron generated LTP of the Str-EP GABAergic synapse on a neighboring neuron (baseline:101 ± 5%; after:138 ± 15%, *N* = 10, Figure 4C, *p* = 0.004).

### Dopamine Modulates eCB Induced Plasticity

**Figure 5:**
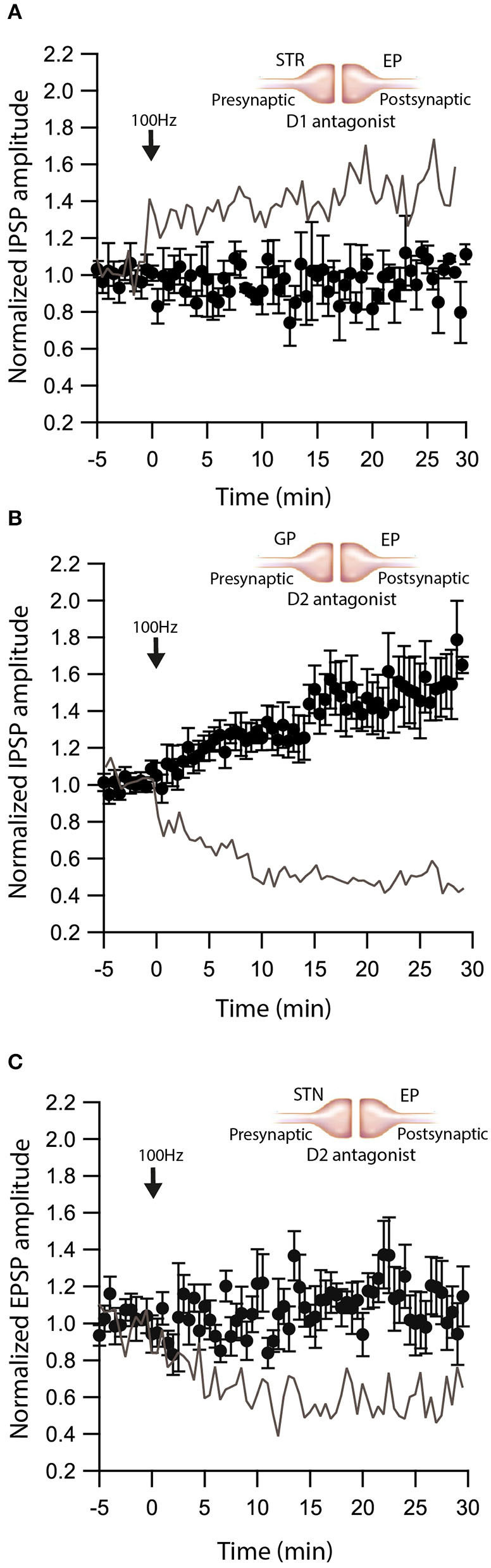
Dopamine modulates eCB induced plasticity. **(A)** Blocking D1 receptors blocks LTP at Str-EP synapses. Post-HFS stimulation of 100 Hz for 10 s in the presence of D1 antagonist at Str-EP synapse (*n* = 4) compared to control condition from Figure 2 in gray. Normalized IPSP amplitudes (normalization to the mean amplitude of IPSP recorded during the baseline recordings) are plotted against time. Arrow indicates the time of induction of the depolarization protocol, and error bars represent the SEM. **(B)** Blocking D2 receptors blocks LTD and reveals LTP at GP-EP synapses. Post-HFS stimulation of 100 Hz for 10 s generated LTP in the presence of a D2 antagonist at the GP-EP synapses (*n* = 7) compared to control condition from Figure 1 in gray. **(C)** Blocking D2 receptors blocks LTP at STN-EP synapses. Post-HFS stimulation of 100 Hz for 10 s in the presence of a D2 antagonist at STN-EP synapses (*n* = 10) compared to the control condition from Figure 3 in gray.

We have shown that dopamine modulates GABAergic inputs to the EP (Lavian et al., 2017). D1Rs modulate GABAergic input from the Str to the EP (Lavian et al., 2017), whereas D2Rs modulate GABAergic input from the GP to the EP. As dopamine interacts with eCBs in other regions of the basal ganglia, we tested the hypothesis that dopamine modulates eCB induced plasticity in the EP. We repeated the experiments described in Figures 1–3 in the presence of dopamine antagonists. We measured the change in Str-EP synapses caused by post-HFS (repeating the experiments shown in Figure 2A) in the presence of R-SCH23390 (10 μM), the D1R antagonist. Under these conditions, post-HFS for 10 s at 100 Hz did not generate LTP (baseline:100 ± 8%; after:96 ± 11%, *N* = 5, Figure 5A, *p* = 0.008) compared to the response of Str-EP synapses (Figure 2A). Then, we measured the change in GP-EP synapses caused by post-HFS (repeating the experiment shown in Figure 1A) in the presence of the D2R antagonist, sulpiride (3 μM). These conditions resulted in LTP of the GP-EP synapse (baseline:101 ± 5%; after:137 ± 14%, *N* = 7, Figure 5B, *p* = 0.002) instead of LTD (Figure 1A). It has been shown that D2Rs modulate glutamatergic input to the EP. Indeed, 3 μM of sulpiride blocked the LTD (baseline:103 ± 7%; after:103 ± 12%, *N* = 10, Figure 5C, *p* = 0.002) that we observed in the experiment without the blocker (Figure 3A).

**Figure 6:**
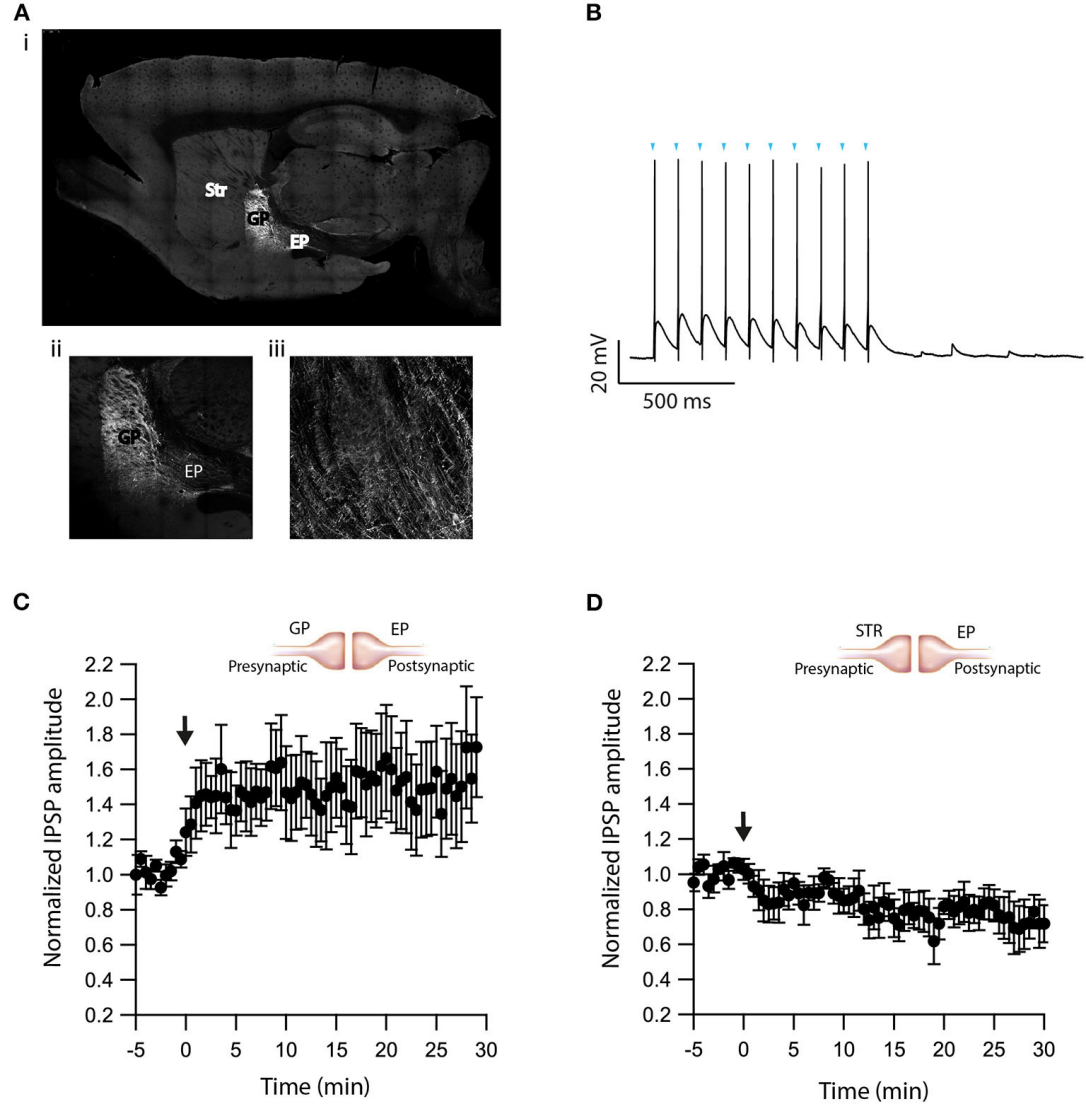
Optogenetic verification of eCB induced plasticity. **(A)** Sagittal brain slice image containing the GP (the injected zone), the EP (the recording zone), and adjacent structures. ChR2-YFP expression was observed in infected cells in the GP and in axons terminals in the EP. The Image was magnified x10 (i), x20 (ii, iii). **(B)** Whole-cell recording from a ChR2 expressing GP neuron illuminated with a 10 Hz light pulse train. The onset of light stimulation is indicated by inverted triangles. The EP neuron‘s membrane potential was set approximately to −60 mV by injecting positive or negative current (~0–20 pA). **(C)** Post-HFS stimulation of 100 Hz for 10 s induced LTP at GP-EP synapses (*n* = 6). Normalized IPSP amplitudes (normalization to the mean amplitude of IPSP recorded during the beginning of baseline recordings) are plotted against time. The arrow indicates the time of depolarization induction protocol, and error bars represent the SEM. **(D)** Post-HFS stimulation of 100 Hz for 10 s generated mild LTD in the presence of a D2 agonist at GP-EP synapses (*n* = 4).

The experiments presented in Figure 5 demonstrate that dopamine modulates eCB induced plasticity in the EP. However, the source of dopamine is unclear. In slice experiments, dopamine release in the EP can result from spontaneous firing of dopaminergic neurons, or non-specific electrical stimulation generating action potentials in dopaminergic axons in upstream BG regions. To differentiate between these possibilities, we performed additional experiments using optogenetics. We injected adeno-associated virus (AAV) encoding a fusion of channelrhodopsin-2 and enhanced yellow fluorescent protein (ChR2-YFP) into the GP of LE-Tg (DRad2-icre) rats (Figure 6Ai). We observed ChR2-YFP expressing somata in the GP (Figure 6Aii) but only axonal projections in the EP (Figure 6Aiii) confirming the localization and specificity of the AAV infection. The GP neuron's firing was locked to individual light pulses within a stimulation train, confirming ChR2-YFP expression in the cell body (Figure 6B). Next, we optogenetically stimulated neurons in the GP while performing whole-cell recordings in the EP. In the presence of CNQX and APV, brief optical stimulation to the GP generated inhibitory synaptic responses in EP neurons. Following a 5 min control period, a 10 s post-HFS at 100 Hz was applied to the soma of the neuron in the EP, generating LTP (baseline:103 ± 3%; after:148 ± 23%, *N* = 6, Figure 6C, *p* = 0.0770)—similar to the LTP produced with electrical stimulation and D2R blocked, thus mirroring the effect observed using electrical stimulation. Bath application of the D2R agonist, Quinpirole (5 μM), produced a small LTD (baseline:101 ± 5%; after:83 ± 9%, *N* = 4, Figure 6D, *p* = 0.02). These experiments suggest that in slice experiments, in addition to the release of GABA, electrical stimulation of the GP produced dopamine release from dopaminergic axons.

### Dopamine and eCBs Modulate EP Firing

**Figure 7:**
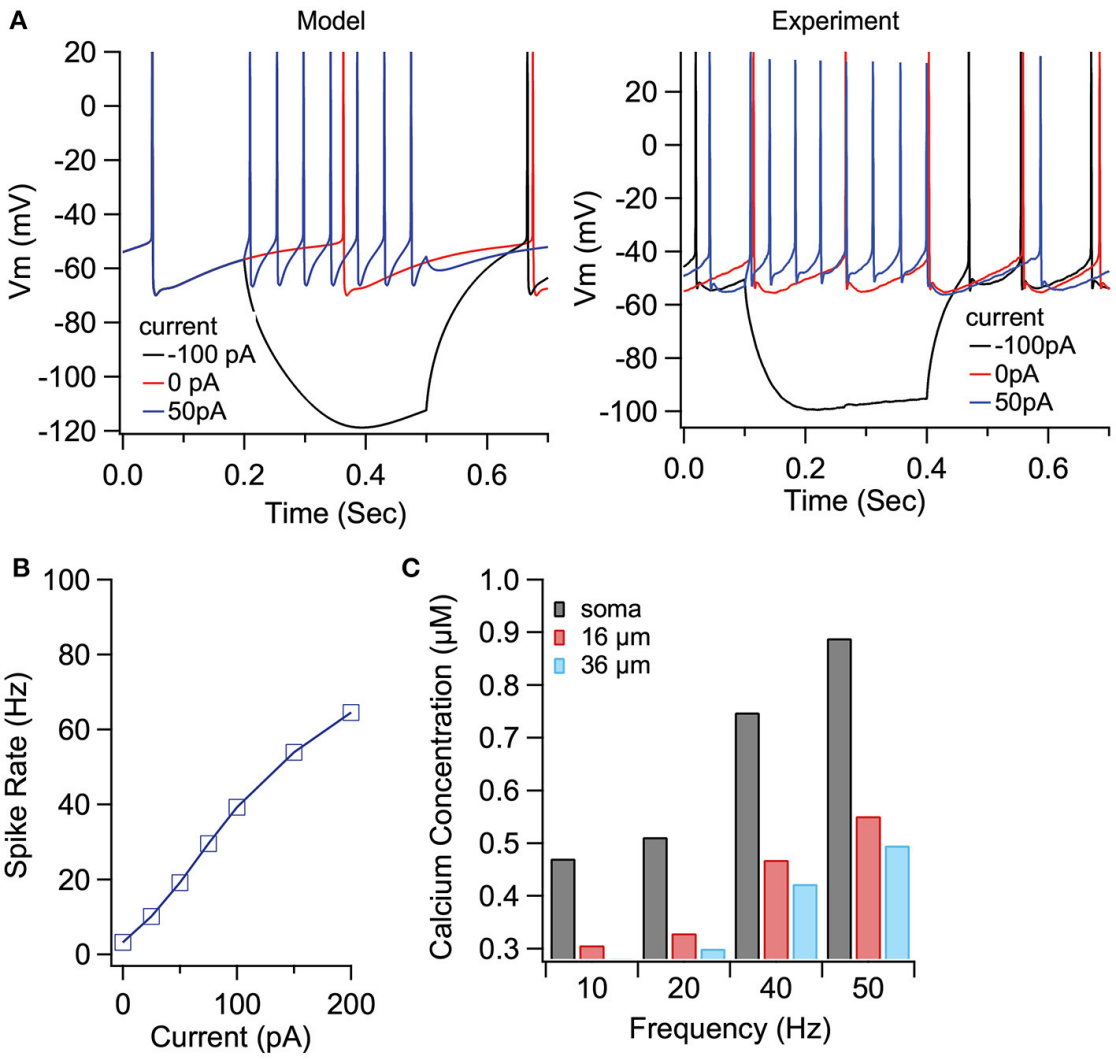
Optimized compartmental model for a neuron in the EP. **(A)** Parameter optimization to EP neuron data produces multi-compartmental EP neuron model with realistic spiking and rectification characteristics. **(A)** Sample traces of EP neuron model (left) and data (right) showing spontaneous activity and response to hyerpolarizing current injection. **(B)** Firing frequency vs. current injection is similar to that measured experimentally. **(C)** Calcium concentration vs. distance from soma and firing frequency is similar to that measured experimentally.

**Figure 8:**
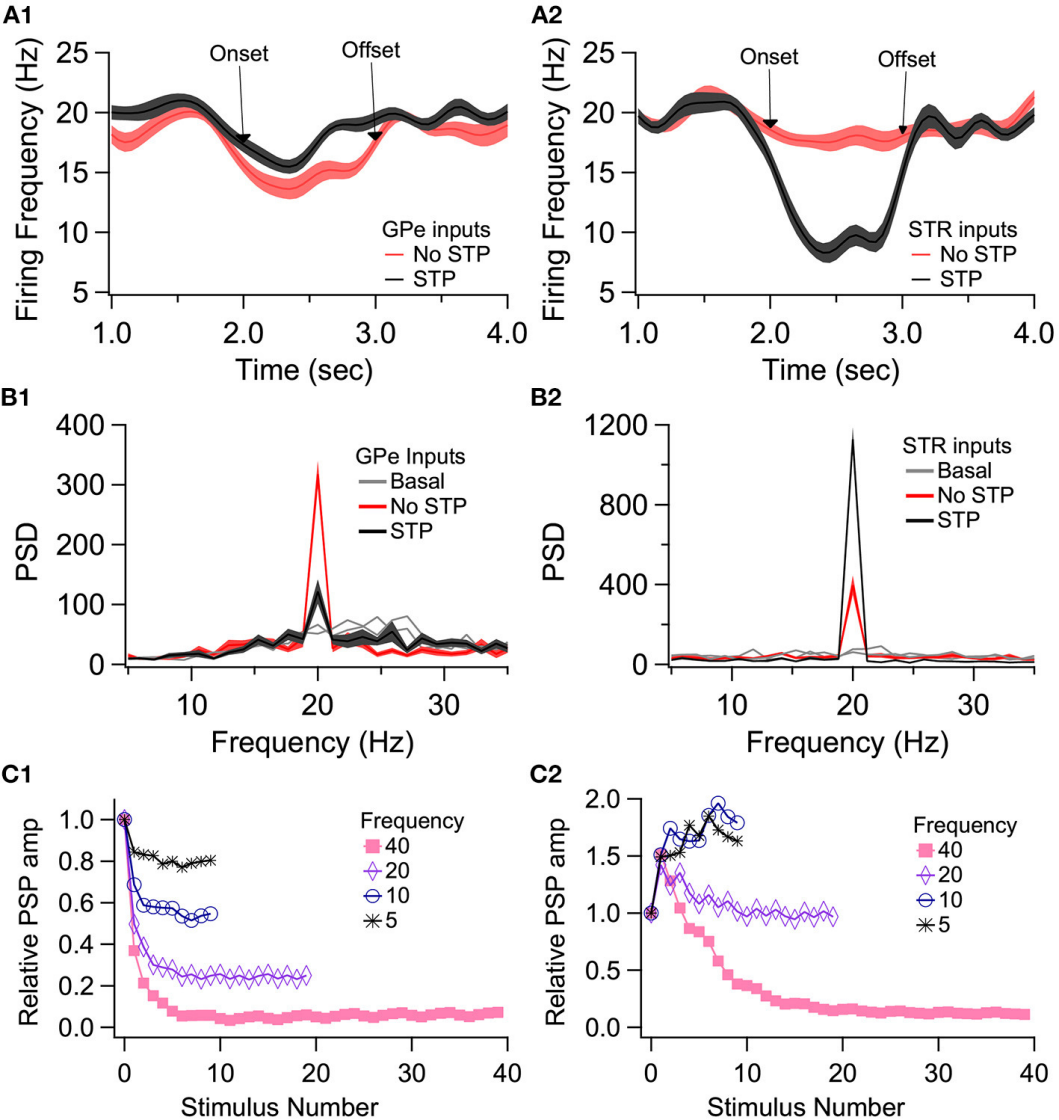
Effect of short term plasticity (STP) on EP firing frequency and phase locking. EP neurons were simulated in response to log normally distributed GPe (18 Hz), SPN (4 Hz), and STN (18 Hz) synaptic input, with an additional 20 Hz train from either GPe **(A1, B1)** or Str **(A2, B2)** delivered between 2 and 3 s (marked by arrows labeled onset and offset). **(A)** Effect of STP on EP firing frequency. STP reduced the effect of 20 Hz GPe inputs on EP firing and enhanced the effect of 20 Hz Str inputs on EP firing. Shading shows +/– 1 SEM. **(B)** Amplitude of the PSD peak at 20 Hz (PSD calculated from the data segment between 2 and 3 s) shows that STP reduced phase locking to GPe inputs but increased phase locking to Str inputs. Basal shows the PSD measured from the same simulations using data segments from 1–2 to 3–4 s. **(C1, C2)** The STP implemented in the model produces similar frequency dependence of PSP amplitude as recorded experimentally.

To demonstrate the role of eCB dependent synaptic plasticity in information processing, we performed simulations of EP neuron activity in response to simultaneous STN, GPe, and Str synaptic inputs. We created a multi-compartmental EP neuron model (Figure 7) by using automatic parameter optimization to adjust the conductance of fast and slow sodium currents, a fast and slow transient potassium current, Kv2 (non-inactivating), and Kv3 (inactivating) potassium channels, small conductance, and big conductance calcium-activated potassium channels, two hyperpolarization-activated cyclic-nucleotide gated (HCN) channels, and one high voltage-activated calcium channel. Figure 7A shows that the spontaneous firing of the EP neuron model and the typical sag in response to hyperpolarizing current injection (Figure 7A-left) resembles that of the recorded neuron (Figure 7A-right). The frequency-current injection curves (Figure 7B) and calcium concentration (Figure 7C) matched experimental data. Using this data-driven model, we assessed the effect of short-term plasticity of Str or GPe inputs to the EP. We performed simulations using short term plasticity (STP) equations derived from fitting experimental data (Lavian and Korngreen, 2016, 2019). Then we simulated the response to 20 Hz stimulation in the presence of log-normally distributed GPe (29 Hz), SPN (4 Hz—to efficiently model the massive convergence from Str to EP, each input train represents four trains firing at 1 Hz) and STN input (18 Hz), both with and without STP of the 20 Hz inputs (STP was always present for the log-normal inputs to maintain ~20 Hz EP neuron firing frequency). First, we measured the effect of STP on EP firing frequency. Figures 8C1,C2 shows that the frequency dependence of STP on single synaptic inputs matches that recorded experimentally. Figure 8A1 shows that 20 Hz GPe inputs produce a 25% reduction in EP firing (No STP) but that this reduction is weaker and more transient with STP. In contrast, Figure 8A2 shows that without STP, Str inputs have a non-significant effect on EP neuron firing, but that with STP the effect of Str inhibition increases, with 50% decrease in EP firing after 300 ms. We further evaluated phase locking of the EP neurons to the 20 Hz input by plotting the power spectral density (PSD). Figures 8B1,B2 shows a peak in the PSD at 20 Hz, representing regular 20 Hz firing in the presence of the 20 Hz input (compared to basal—the absence of 20 Hz input). The peak at 20 Hz is greater in the absence of STP for GPe inputs, and in the presence of STP for Str inputs.

**Figure 9:**
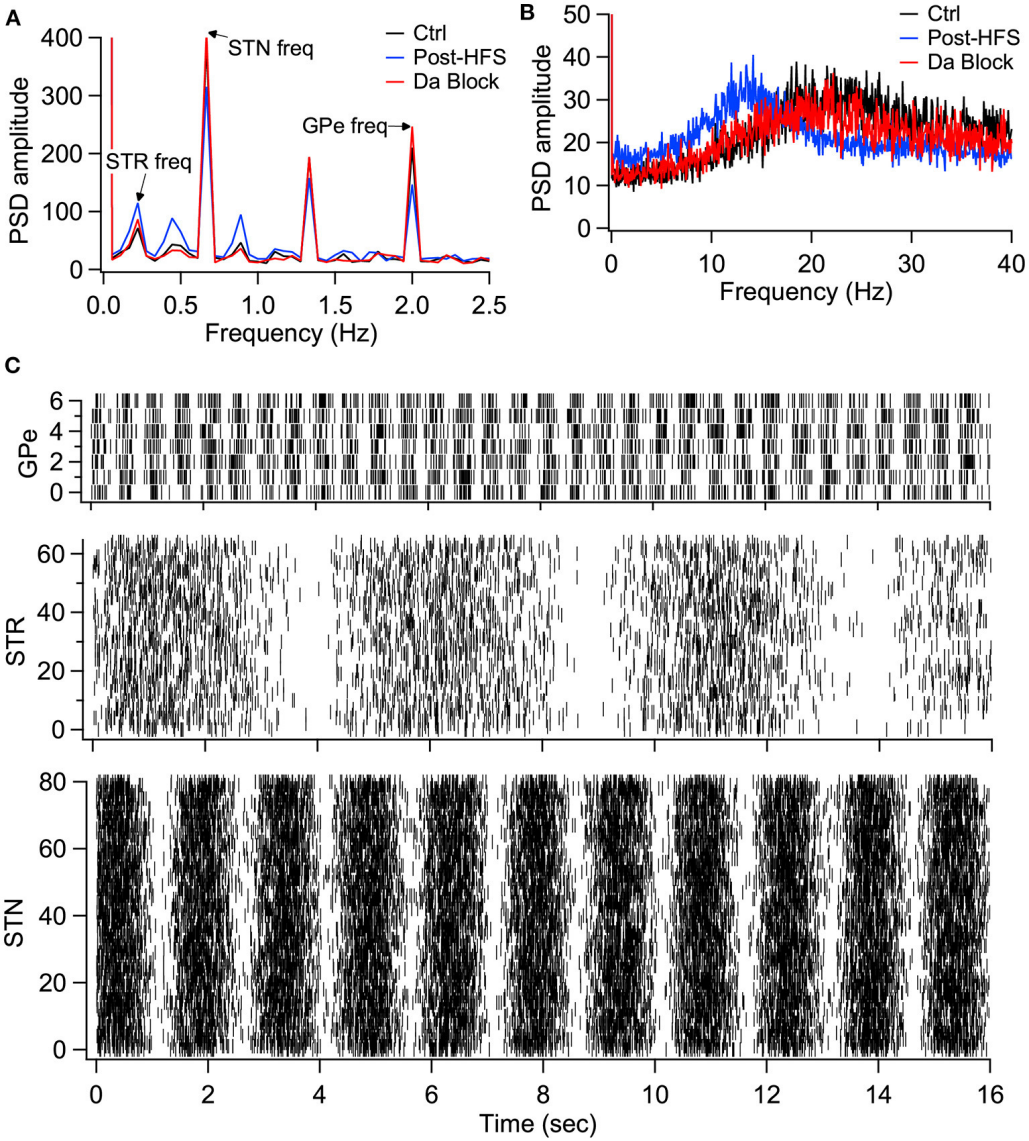
Model simulations demonstrate the eCB dependent synaptic plasticity enhances information transmission from the striatum and reduces energy in the β band. **(A)** Simulations in response to oscillatory synaptic inputs reveals that Post-HFS enhances energy at the Str frequency while reducing energy at GPe and STN frequencies. *N* = 15 trials. **(B)** Simulations in response to log normally distributed synaptic inputs demonstrates that Post-HFS shifts energy into the β-band, whereas blocking dopamine during Post-HFS does not change energy in the β-band. *N* = 15 trials. **(C)** Raster plots of synaptic inputs for 1 trial of simulations in **(A)**. Oscillation frequency of 0.2 Hz (Str), 0.6 Hz (STN), and 2 Hz (GPe) modulates the mean firing frequency of synaptic inputs. Note that mean firing frequency of synaptic inputs was 4 Hz (Str), 18 Hz (STN), and 29.3 Hz (GPe) for both oscillatory **(A)** and log normally distributed **(B)** inputs.

We then evaluated the effect of long-term plasticity of Str and GPe inputs. We simulated the response to Str, GPe and STN synaptic inputs under three different conditions: Control, synaptic strength resulting from the post-HFS protocol, and synaptic strength resulting from the post-HFS protocol with dopamine blocked. Mean firing frequency of these synaptic inputs was similar to that measured experimentally (Kita and Kita, 2011; Wilson and Bevan, 2011), thus Str inputs fired at 4 Hz, GPe inputs at 29 Hz, and STN inputs at 18 Hz. To demonstrate how synaptic plasticity changes information transmission, we modulated the mean firing frequency (spike trains generated using an inhomogeneous Poisson process), with a different oscillation frequency for STN, GPe and STR (Figure 9C). The information transmitted by the EP is represented as the amplitude of the PSD at the oscillation frequency for each structure. Figure 9A shows that synaptic plasticity (Post-HFS) increases the Str (direct pathway) information while reducing the GPe and STN information, but this enhancement is eliminated with dopamine blocked. We also performed simulations using log-normally distributed synaptic inputs, which captures the long tailed inter-spike-interval distributions observed *in vivo* (Kita and Kita, 2011). Figure 9B shows that the post-HFS protocol decreases energy at 20 Hz (β frequency), whereas post-HFS with dopamine blocked has enhanced energy at 20 Hz.

## Discussion

Here we present results supporting a new integrative role for dopamine and eCBs in modulating BG output. Using electrophysiology, optogenetics, and modeling, we presented evidence suggesting interdependence between the direct, indirect, and hyperdirect afferents to the EP. We demonstrated that eCB modulates synaptic strength of the indirect pathway (Figure 1), the direct pathway (Figure 2), and glutamatergic (Figure 3) inputs to the EP, and that dopamine modulates eCB induced plasticity of all inputs to the EP (Figure 5). We also showed that eCBs diffuse, modulating synaptic strength of neighboring EP neurons (Figure 4). Thus, despite the lack of axon collaterals, information is transferred between neurons in the EP *via* endocannabinoid diffusion. Our numerical simulations demonstrate that the combination of STP and eCB mediated long-term plasticity enhances the transmission of direct pathway information while reducing indirect and hyper-direct pathway information. Thus, our results propose that the passage of BG information through the bottleneck of the EP allows modulation of the information using small neuronal populations. Our data suggest that this cellular mechanism controls the balance between direct and indirect pathway information in the EP output.

eCB mediated synaptic plasticity is critically involved in neural functions ranging from homeostasis to cognition (Katona and Freund, 2012). Behavioral adaptations rely on changes in synaptic strength and the prevalence of eCB-mediated long-term depression (eCB-LTD) at synapses (Castillo et al., 2012). eCBs have been identified as triggers for short and long-term plasticity at synapses throughout the brain. There is a notable expression of CB1Rs in the basal ganglia, mainly in the Striatum, GP, EP, and SNr (Herkenham et al., 1990). Activation of the CB1R modulates LTD and also short-term plasticity (Llano et al., 1991; Pitler and Alger, 1992; Morishita and Alger, 2000; Kreitzer and Regehr, 2001; Ohno-Shosaku et al., 2001; Wilson and Nicoll, 2001). This modulation of short-term plasticity appears in several basal ganglia regions including the striatum (Kreitzer and Malenka, 2005; Freiman et al., 2006; Narushima et al., 2006a,b, 2007; Yin and Lovinger, 2006; Centonze et al., 2007; Uchigashima et al., 2007; Maccarrone et al., 2008), GP (Engler, 2005), SNr (Yanovsky et al., 2003; Engler, 2005; Szabo et al., 2006), SNc (Szabo et al., 2006), and NAc (Rancz and Ha, 2006). Furthermore, eCB induced LTD has been reported in several brain regions such as the dorsal striatum (Kreitzer and Malenka, 2005, 2007; Ronesi and Lovinger, 2005; Wang et al., 2006), NAc (Robbe et al., 2002; Mato et al., 2008), the cortex (Panikashvili et al., 2005; Nevian and Sakmann, 2006; Lafourcade et al., 2007), the cerebellum (Tzounopoulos et al., 2007), amygdala (Marsicano et al., 2002; Azad et al., 2004), and the hippocampus (Chevaleyre and Castillo, 2003, 2004; Chevaleyre et al., 2007).

Here we demonstrated that glutamatergic input to the EP exhibit eCB-LTD that is induced by post-synaptic firing. The eCB induced LTD reported here shares similarities with striatal LTD. HFS of corticostriatal glutamatergic inputs to SPNs is known to induce LTD that requires post-synaptic Ca^2+^ elevation and leads to a decrease in the probability of glutamate release (Calabresi et al., 1992, 1994; Choi and Lovinger, 1997; Kreitzer and Malenka, 2005). Striatal LTD is dependent on group I mGluRs and L-type Ca^2+^ channels. Ca^2+^ elevation by an LTD induction protocol induces eCB release. Results presented here suggest a calcium dependent LTD. The depression level of the STN-EP synapses depends on the frequency of the APs in the EP neurons. This matches the AP frequency dependence of Ca^2+^ concentration in the soma and the dendrite of the EP neurons (Gorodetski et al., 2018), suggesting that the observed eCB-LTD is indeed calcium-dependent. Post-synaptic release of eCBs is sufficient to induce CB1R-mediated depression at GABAergic synapses (Adermark et al., 2009). Similarly, the synaptic depression reported here is blocked by AM-251, implicating CB1R in this LTD. On the other hand, in the direct pathway, Str-EP synapses exhibit eCB-LTP that also depends on the frequency of the APs and is blocked by CB1Rs antagonist. An eCB-LTP also has been observed in the dorsal striatum (Cui et al., 2015). These similarities suggest that HFS induced by high frequency firing of STN neurons could induce synaptic plasticity.

After identifying the eCBs effect on the EP pathways, we demonstrated that dopamine modulates the eCB induced plasticity (Figure 5). Striatal evoked IPSCs exhibit short-term facilitation that is modulated by dopamine *via* D1LR, which are co-localized with striatal axon terminals (Lavian et al., 2018). Furthermore, GP evoked IPSCs that show short-term depression are modulated by dopamine *via* D2LR, which are co-localized with GP axon terminals (Lavian and Korngreen, 2016; Lavian et al., 2017). In the current study, we showed, both pharmacologically and optogenetically, that eCB effects require D1R (at Str-EP synapses) or D2R (at STN-EP synapses). These results indicate that DA modulates the generation of eCB-LTD by synaptic activity and comply with previous work implicating dopamine modulation of state-dependent eCB release in the striatum (Kreitzer and Malenka, 2005, 2007). However, in the GP-EP synapses, the block of D2R resulted in LTP instead of LTD, which differs from corticostriatal synapses (Xu et al., 2018), where eCB-LTP *requires* D2Rs. Our observations, suggests that these G protein coupled dopamine receptors are interacting with eCB production pathways, as has been described in the striatum, though the molecule mechanisms in the EP have not yet been characterized.

Finally, we demonstrate that eCBs diffuse and modulate synaptic plasticity in neighboring neurons (Figure 4). The diffusion of eCBs implies that all three types of synapses likely undergo plasticity at the same time. Moreover, nearby neurons that do not fire at high frequency may still experience modulation of synaptic inputs. These results suggest that the EP is not only a feedforward nucleus (Parent et al., 2000), despite containing no axon collaterals, but also has lateral interactions. In other words, high frequency STN inputs could trigger EP firing and heterosynaptic plasticity of GP or Str synapses, modulating the entire recombination process of direct and indirect pathways to the EP.

We created a data-driven model of EP neurons, and measured the response to *in vivo* like inputs to Str, STN, and GPe synapses simultaneously, to evaluate the functional effect of the simultaneous plasticity of all three synapses, as might occur with high frequency STN input and eCB diffusion. Our modeling results show that eCB mediated long term plasticity enhances information transmission from the direct pathway, compared to indirect and hyper-direct pathways, confirming that eCB-mediated plasticity controls the balance of information transmission through the basal ganglia, and thus may directly influence decision making behavior. We further show that the eCB-mediated enhanced direct pathway information is eliminated when dopamine is blocked, confirming that dopamine is critical for controlling basal ganglia output directly. Many models of the basal ganglia have evaluated the role of dopamine in action selection. In most of these models (Humphries et al., 2006; Leblois et al., 2006; Lindahl and Kotaleski, 2016), dopamine influences direct vs. indirect pathway information by its effect on striatal activity—either excitability or corticostriatal synaptic plasticity. One model implemented short term depression of inputs to both GPe and SNr (another BG output region) (Lindahl and Kotaleski, 2016); however, the contribution SNr input modulation to action selection was not evaluated. Several network models evaluated the effect of dopamine on GPi (primate analog of EP) oscillatory firing, to investigate mechanisms underlying Parkinson's beta oscillations and normalization by DBS (Hahn and McIntyre, 2010; Humphries and Gurney, 2012; Kumaravelu et al., 2016). In these models, dopamine modulates striatal or GPe neurons or synapses, and beta oscillations in the GPi are a readout of basal ganglia network state. In contrast, our model revealed a direct effect of dopamine on beta oscillations. Specifically, the model exhibits a small increase in power at beta frequency, produced solely by dopamine mediated changes in synaptic plasticity of EP neurons. Thus, this suggests that lack of dopamine in the GPi can contribute to the production of beta oscillations in Parkinson's.

Overall, our results suggest the EP has two modes of operation, based on the anatomical and functional polarity of EP neurons. In one mode, GABAergic inputs from the GP act, due to their short-term depression kinetics and high baseline firing rates, as somatic shunting inhibition delivering an almost constant inhibition to the soma (Bugaysen et al., 2013; Lavian and Korngreen, 2019). The second mode occurs periodically during brief periods of striatal activation by cortical and thalamic inputs. The consequent amplification of dendritic inputs produces phase locking of EP firing to that of the Str (Lavian and Korngreen, 2016; Lavian et al., 2017), overcomes the somatic shunt, and allows short bursts of Str firing to affect the firing of neurons in the EP. It is possible to visualize GABAergic neurons in the EP as having a somatic obstacle surmounted by a dendritic amplifier. It is worth noting that neurons in the EP fire spontaneously over a wide range of frequencies. Figures 1D, 2D, 3D show a functional relationship between the firing frequency and the change to synaptic strength. Thus, the spontaneous firing of EP neurons probably modulates synaptic strength continuously. We induced plasticity using a 10-s train of action potentials ranging between 10 and 100 Hz covering the entire range of firing for rodent EP neurons, with 100 Hz being an upper boundary for EP firing rate in rodent. Furthrmore, it is likely that STN inputs would be able to produce synaptic plasticity at lower firing frequencies because the concomitant activation of group 1 mGluRs would facilitate eCB production (Covey et al., 2017).

**Figure 10:**
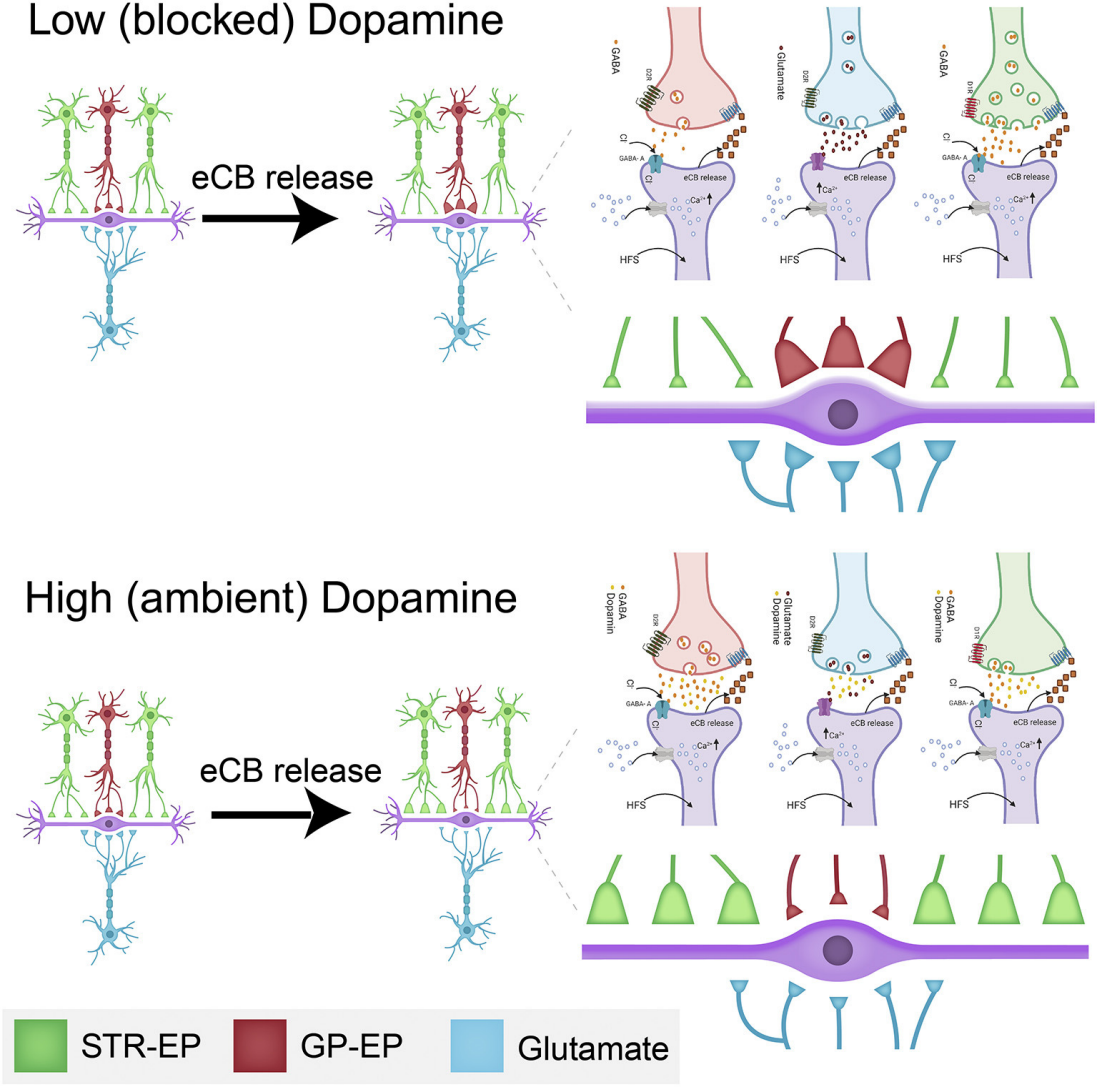
Schematic drawing of the dopamine-eCB induced synaptic dynamics in the EP. The top drawing displays the changes schematically to synaptic strength at low (or blocked) dopamine levels. The bottom illustration shows the changes schematically to synaptic strength at high (or ambient) dopamine levels. The changes to synaptic strength are depicted by the relative changes to the size of individual synaptic terminals in the figure and enlarged on the right. Some of the biochemical mechanisms involved in plastic changes described in this manuscript are shown in each panel. These detailed drawings were made with Biorender.com.

The observation that dopamine interacts with eCBs to modulate all synaptic inputs to the EP has highly significant implications. In the presence of dopamine, an increase in EP firing (probably due to glutamatergic input from the STN) lowers the somatic barrier (eCB-LTD in GP-EP synapse) while boosting the dendritic amplifier (eCB-LTP in Str-EP synapse). In other words, a burst of dopamine plus STN inputs will increase the weight of input from the direct pathway while lowering that from the indirect pathway (Figure 10); thereby allowing Str to control EP firing for a longer duration than predicted by STP alone. Moreover, increased EP firing lowers the weight of glutamatergic input to the EP, further increasing the control of EP output by input from the striatum. The concomitant release and diffusion of eCBs may modulate neighboring synapses on the same neuron or on nearby neurons. This spatial effect may lower the somatic barrier even more while boosting the dendritic amplifier for a population of EP neurons. The net result of this cellular mechanism for “*selection”* is to determine whether information from the direct or indirect pathway will dominate EP output. These results transform the prevailing view of the entopeduncular nucleus as a feedforward “relay” nucleus to an intricate control unit, which may play a vital role in the process of action selection.

## Data Availability Statement

The raw data supporting the conclusions of this article will be made available by the authors, without undue reservation.

## Ethics Statement

The animal study was reviewed and approved by Bar-Ilan Institutional Animal Care and Use Committee.

## Author Contributions

AK and LG designed the study, performed the experiments, and analyzed the data. KB implemented the model simulations and analyzed the modeling results. AK, LG, and KB drafted and revised the manuscript. All authors read and approved the final version of the manuscript for publication.

## Conflict of Interest

The authors declare that the research was conducted in the absence of any commercial or financial relationships that could be construed as a potential conflict of interest.

## Abbreviations

STN: subthalamic nucleus
EP: entopeduncular nucleus
SNr: substantia nigra reticulate
LTD: long-term depression
CB1: endocannabinoid 1
GP: globus pallidus
ACSF: artificial cerebrospinal fluid
DMSO: Dimethyl sulfoxide
ROI: regions of interest
F: Fluorescence
LTP: long term potentiation
AP: Action potential
SPNs: spiny projection neurons.
